# Preparation and Photocatalysis of CuO/Bentonite Based on Adsorption and Photocatalytic Activity

**DOI:** 10.3390/ma14195803

**Published:** 2021-10-04

**Authors:** Cuina Yang, Hongfa Xu, Jicun Shi, Zhifeng Liu, Lei Zhao

**Affiliations:** 1 State Key Laboratory of Disaster Prevention & Mitigation of Explosion & Impact, Army Engineering University of PLA, Nanjing 210007, China; Yangcuina213@126.com; 2 School of Civil Engineering and Architecture, Xinxiang University, Xinxiang 453003, China; jicun.shi070@xxu.edu.cn (J.S.); zhaolei197604@126.com (L.Z.); 3 School of Materials Science and Engineering, Tianjin Chengjian University, Tianjin 300384, China; tjulzf@163.com

**Keywords:** CuO, bentonite, CuO/bentonite composite, adsorption, photocatalysis

## Abstract

A CuO/bentonite composite photocatalyst was prepared to fully utilize the adsorption capacity of bentonite and the photocatalytic activity of CuO. CuO and bentonite were chosen as a photocatalyst due to the excellent optical property of CuO and large specific surface area of bentonite, together with their high stability and low production cost. The sample was characterized by XRD, SEM, and BET. The effects of several factors on degradation process were investigated such as dosage of H_2_O_2_, irradiation time, pH of the solution, and dosage of catalyst. The optimum conditions for decolorization of methylene blue solution by CuO/bentonite were determined. Under optimal conditions, the decolorization efficiency of methylene blue by a 1.4% CuO/bentonite (400 °C) composite photocatalyst under visible irradiation at 240 min reached 96.98%. The degradation process follow edpseudo-second-order kinetics. The photocatalytic mechanism is discussed in detail. This composite structure provides a new solution to the cycle and aggregation of the photocatalyst in water.

## 1. Introduction

In recent years, with the wide application of dyes in industry, a large amount of wastewater has been polluted by colorants. Thus, environmental and energy problems have become increasingly prominent. Dyes are relatively poorly biodegradable substances, which can cause the accumulation of these pollutants in the environment. It should also be noted that some dyes can be toxic or carcinogenic. The massive discharge of organic pollutants seriously endangers human health and is difficult to remove by conventional methods. Thus, it is crucial to find modern methods for dye removal based on natural or waste materials [1]. Among the various techniques used to remove dyes, one of the most popular is adsorption technology. Although adsorption is an effective and straight forward method, the regeneration of the adsorbent increases the operation cost. One of the alternatives to adsorption is the photocatalytic degradation process. Photocatalysis is considered waste-free and can additionally use sun rays as a natural source of energy. Photocatalytic technology based on semiconductor materials provides a new way to solve energy and environmental problems [2,3,4]. Semiconductor photocatalysts can obtain enough photonenergy (equal to or greater than the bandgap energy of semiconductor) to generate photogenerated electron–hole pairs. Photogenerated holes of the semiconductor under visible irradiation can drive complex chemical reactions at the surface for organic dye degradation in wastewater [5,6,7]. At present, titanium dioxide (TiO_2_) is the most popular material in the photocatalytic process. Due to its improved photocatalytic performance, titanium dioxide is also widely used in the form of nanoparticles [8]. Another successful photocatalyst is zinc oxide (ZnO) nanoparticles. The advantages of nano zinc oxide are environmental stability and its low cost compared with other nano oxides. The main problem with similar titanium and zinc oxides is the high bandgap value of 3.2–3.3 eV, which means that they can only absorb UV light, which occupies 4% of total sunlight, limiting their large-scale application [9,10]. Therefore, researchers are committed to studying visible-light-driven photocatalysts, such asBi_2_WO_6_, CuO, and BiOBr [11,12,13]. Among these, CuO is a new p-type semiconductor material with a small particle size and large specific surface area. CuO has a relatively narrow bandgap (1.2–1.5 eV) and excellent photocatalytic performance, making full use of visible irradiation. At present, nano CuO is widely applied in the catalysis [14,15], sensing [16], and battery materials fields [17].

However, it cannot be ignored that TiO_2_, ZnO, and CuO are easy to agglomerate and difficult to recover in water. Thus, many efforts have been made to immobilize the photocatalysts on some inert carriers, such as activated carbon [18], silica [19], zeolite [20,21,22], and bentonite [23]. Li [24] used activated carbon-loaded nano CuO to treat dye wastewater, and the removal rate of COD reached 84.6%. Nezamzadeh-Ejhieh et al. [25] used zeolite to load nano CuO, and the decolorization efficiency of methylene blue solution reached 94%. Liu et al. studied the photocatalytic activity of a TiO_2_/zeolite photocatalyst under UV irradiation and a CuO/zeolite photocatalyst under visible irradiation for MB in water [26,27]. Bentonite is a 2:1 layered silicate mineral composed of two layers of silica tetrahedron and one layer of alumina octahedron, which has expansibility [28,29]. Compared with other clay minerals, bentonite has excellent adsorption capacity and cation exchange sites on its interlayer surface, outer surface, and edge [30]. Additionally, many studies have shown that bentonite is a suitable substrate for the synthesis of composite photocatalysts with photocatalytic activity [31,32,33]. The study of Ma et al. on silver phosphate dispersed on bentonite showed that the photocatalytic degradation activity of rhodamine B under visible light was significantly improved [34]. Combining the adsorption and photocatalytic process can better remove dyes in solution and achieve a higher value of q_max_ (maximum adsorption capacity). The difference in the amount of dye extracted from the solution during the two processes is due to photocatalytic degradation under UV irradiation [23]. However, there are few reports about the use of a CuO/bentonite composite for the degradation of MB in water.

To solve the problems of nano CuO, such as easy agglomeration and difficult recovery, a new type of CuO/bentonite composite based on adsorption and photocatalytic activity was prepared in this study. Due to its excellent optical properties, unique porous structure, and stability, the composite of CuO and bentonite fully exerted the adsorption capacity of bentonite and the photocatalytic activity of CuO. Methylene blue (MB) solution was used as a target pollutant, and visible irradiation was simulated; the photocatalytic activity of CuO/bentonite composite was evaluated by the fading degree of MB in water under visible irradiation. The photocatalytic performance of the CuO/bentonite composite was characterized, and the optimal amount of CuO loaded on bentonite was determined. The adsorption kinetics of CuO/bentonite was explored, and the photocatalytic mechanism of CuO/bentonite composite was discussed in detail.

## 2. Experimental

### 2.1. Materials

Bentonite was purchased from Tianjin Guangfu Fine Chemical Research Institute (Tianjin, China). Copper nitrate (C99.0–102.0%), hydrogen peroxide (30%), sodium hexametaphosphate, sodium carbonate, sodium hydroxide (99%), hydrochloric acid (36%), and methylene blue were purchased from Sinopharm Chemical Reagent Co., Ltd. (Shanghai, China) and used as received without further purification.

### 2.2. Preparation of Na-Bentonite and CuO/Bentonite

First, 320 g of the original bentonite was filtered with a 200 mesh sieve, and Na_2_CO_3_ was added simultaneously. The ratio of Na_2_CO_3_ and bentonite was 3% (by weight). Subsequently, the mixture was thoroughly stirred at 25 °C for 1 h and left to stand for 24 h. Then, the slurry concentration was diluted to 15% with sufficient stirring and the impurities at the bottom of the beaker were discarded. This process was repeated several times until there was no solid material at the bottom of the slurry. Finally, the slurry was centrifuged and dried at 105 °C, then ground and sieved through 200 meshes to obtain Na-bentonite.

A quantity of 11.6 g of Na-bentonite was added to 100 mL of 0.02 mol/L Cu(NO_3_)_2_ and stirred for 12 h at room temperature. Afterward, the sample was placed in an oven and dried at 60 °C. Finally, the dried sample was divided into three parts, placed in a muffle furnace, and calcined at temperatures of 300 °C, 400 °C, and 500 °C for 4 h, before cooling to room temperature. After grinding and sieving, CuO accounted for 1.4% of the CuO/bentonite composite photocatalyst which was obtained.

### 2.3. Characterization

The crystalline structure of the samples was analyzed by Rigaku D/max-2500v/peX-ray diffraction (XRD, Rigaku, Tokyo, Japan) with Cu=Kα radiation (λ = 0.154059 nm). Test conditions were as follows: tube voltage 40 kV, tube current 40 mA, scanning range 3°–80°, and scanning speed 8°/min. Scanning electron microscopy (SEM, JSM-7800F, JEOLLtd., Tokyo, Japan) was used to evaluate the morphology information of the samples. BET measurements of samples were made using nitrogen at 77 K as an adsorbed gas on the ASAP 2020 (BET, ASAP2020HD88, Micromeritics Instrument Ltd., Atlanta, GA, USA).

### 2.4. Sorption and Photocatalysis Processes

The photocatalytic performance of the CuO/bentonite composite was evaluated by measuring the decolorization efficiency of methylene blue (MB). A certain amount of composite material was mixed with a 50 mg/L MB solution and stirred in the dark (stainless-steel box for shielding external light) for 30 min to achieve the adsorption–desorption equilibrium of bentonite. Subsequently, a 30 W energy-saving lamp was positioned above the 100 mL beaker (25 cm) to supply visible irradiation. The supernatants were centrifuged and collected at different times (30, 60, 90, 120, 150, 180, 210, and 240 min) under visible irradiation. A UV/Vis spectrophotometer 765 (Shanghai Jinghua Technology Instrument Co., Ltd., Shanghai, China) was used to measure the absorbance of methylene blue aqueous solutions at 664 nm. The decolorization efficiency of MB was calculated using Equation (1).

(1) $$D=\left[(A_{0}-A_{t})/A_{0}\right]\times 100\%,$$

where D is the decolorization efficiency of MB at time t (min), A_0_ is the initial absorbance of MB, and A_t_ is the absorbance value of MB at irradiation time t.

The sorption capacities at a given time (q_t_) and in a state of equilibrium (q_e_) were calculated using Equations (2) and (3) [35].

(2) $$q_{t}=\left(\frac{C_{0}-C_{t}}{W}\right)\times V,$$

(3) $$q_{e}=\left(\frac{C_{0}-C_{e}}{W}\right)\times V,$$

where q_t_ is the weight of adsorbed MB at time t (mg/g), q_e_ is the weight of adsorbed MB at equilibrium (mg/g), C_0_ is the initial concentration of MB (mg/L), C_t_ is the concentration of MB at time t (mg/L), Ce is the concentration of MB at equilibrium (mg/L), V is the volume of solution (mL), and w is the CuO/bentonite weight used in the sorption process (mg).

The kinetic parameters of CuO/bentonite composite for the MB photocatalytic process can be fitted by pseudo-first-order and pseudo-second-order kinetic models [36].

The pseudo-first order model equation is expressed by Equation (4).

(4) $$\ln(q_{e}-q_{t})=\ln q_{e}-k_{1}t,$$

where q_e_ (mg·g^−1^) is the MB adsorption capacity at equilibrium, q_t_ (mg·g^−1^) is the amount of adsorbed MB at time t, k_1_ (min^−1^) is the constant for the model, and t (min) is time.

The pseudo-second order model equation is expressed by Equation (5).

(5) $$\frac{t}{q_{t}}=\frac{1}{k_{2}q_{e}^{2}}+\frac{t}{q_{e}},$$

where q_e_ (mg·g^−1^) is the MB adsorption capacity at equilibrium, q_t_ (mg·g^−1^) is the amount of adsorbed MB at time t, k_2_ (g·mg^−1^·min^−1^) is the constant for the model, and t (min) is time.

## 3. Results and Discussion

### 3.1. XRD Analysis

**Figure 1:**
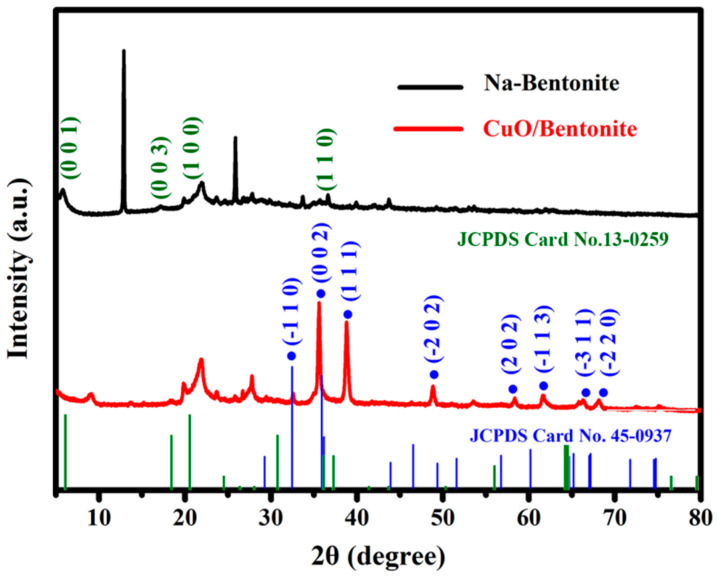
XRD of Na-bentonite and 1.4% CuO/bentonite(400 °C) composite.

XRD patterns of Na-bentonite and the 1.4% CuO/bentonite (400 °C) composite are shown in Figure 1. According to Figure 1, Na-bentonite was strongly characterized by the diffraction peak of montmorillonite (001); a crystal plane appeared at 2θ = 5.88°. The diffraction peak intensity of 1.4% CuO/bentonite (400 °C) decreased significantly and the diffraction angle increased to 9.12°, which was caused by the decrease in crystal plane order and layer spacing of montmorillonite due to the partial entry of Cu^2+^ into the montmorillonite layer of bentonite and the replacement of some hydrated metal ions. The D-value of the (001) diffraction of the Na-bentonite was 15.3 Å, similar to that reported by Andreola [37]. In Figure 1, the peaks located at 17.08°, 19.87°, 21.55°, 27.92°, 28.41°, and 36.31° could be assigned to Na-bentonite [38,39]. When the diffraction angles 2θ were 32.496°, 35.495°, 38.730°, 48.725°, 58.335°, 61.533°, 66.248°, and 68.089°, the corresponding crystal planes were (−110), (002), (111), (−202), (202), (−113), (−311), and (−220), respectively. These are consistent with the standard spectrum of monoclinic CuO in the standard spectrum library (JCPDS 45-0937). The characteristic peaks of montmorillonite 2θ = 19.87°, 21.55°, and 36.31° appeared in 1.4% CuO/bentonite (400 °C). The XRD pattern of 1.4% CuO/bentonite (400 °C) included both CuO and the characteristic peaks of bentonite. The results showed that the prepared sample was a complex of CuO and bentonite. At the same time, the intensity of some diffraction peaks decreased, which may have been due to the combination of CuO and bentonite. In addition, the Scherrer equation was used to calculate the average crystallite size of CuO from the X-ray diffraction patterns using Equation (6) [40].

(6) D=kλ(βcosθ),

where *D* is the crystallite size, *k* is a constant (*k* = 0.943), *λ* is the wavelength (X-ray radiation Cu Kα, *λ* = 0.1540059 nm), *β* is the full width at half maximum (FWHM) of the diffraction peak, and *θ* is the diffraction angle. The crystallite size of CuO was about 33.5 nm, as calculated by the Scherrer equation.

### 3.2. SEM Analysis

**Figure 2:**
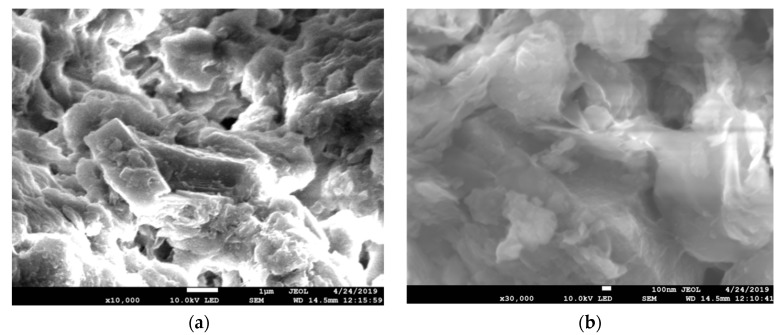
SEM images of (**a**) Na-bentonite, and (**b**) 1.4% CuO/bentonite(400 °C) composite.

SEM images of Na-bentonite and the 1.4% CuO/bentonite (400 °C) composite are shown in Figure 2. It can be seen from Figure 2a that Na-bentonite had an obvious contour, obvious lamellar structure, different sizes of lamellar structure, no flocsor edge burrs, and a smooth outer surface. After loading CuO, as shown in Figure 2b, the morphology of the composite photocatalyst was still lamellar in structure, but the lamellar structure was looser and the surface was rougher. This lamellar structure caused the composite photocatalytic materials to have a large specific surface area, which is conducive to the photocatalytic reaction. Some CuO entered the interlayer of bentonite, while the remainder was loaded onto the surface of bentonite, which is consistent with the XRD analysis.

### 3.3. Photocatalytic Performance

#### 3.3.1. Effect of H_2_O_2_ Dosageon Decolorization Efficiency of MB Solution

**Figure 3:**
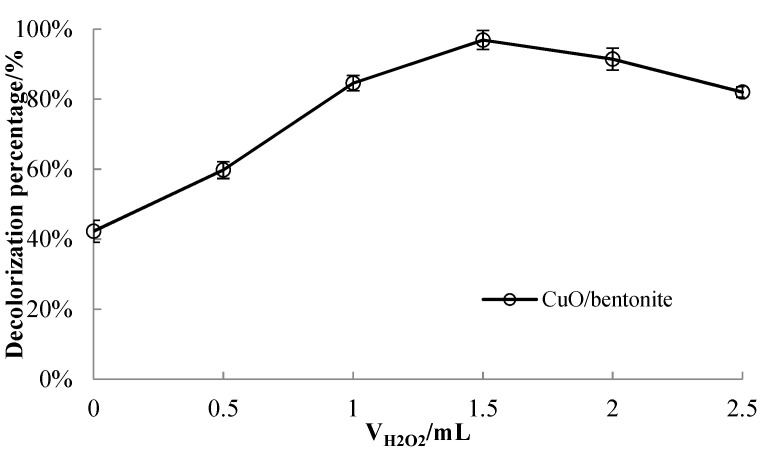
Effect of H_2_O_2_ dosage on decolorization efficiency of the MB solution.

A quantity of 50 mL of the 50 mg/L MB solution was taken and adjusted so that the pH value was 7.0. A quantity of 0.3 g/L of the CuO/bentonite composite was added. They were allowed to react under visible irradiation 240 min at room temperature to investigate the effect of H_2_O_2_ dosage on MB decolorization efficiency. The results are shown in Figure 3.

It can be seen from Figure 3 that, when the amount of H_2_O_2_ was 0 mL, the decolorization efficiency of MB solution by CuO/bentonite was 42.28%. At this time, it was mainly contributed by the adsorption of bentonite. The decolorization efficiency of CuO/bentonite increased with the appropriate addition of H_2_O_2_. When increasing the amount of H_2_O_2_, the decolorization efficiency of MB solution by CuO/bentonite gradually increased. When the amount of H_2_O_2_ was 1.5 mL, the decolorization efficiency reached 96.37%. However, upon further increasing the amount of H_2_O_2_, the decolorization efficiency of MB by CuO/bentonite decreased, which may be because H_2_O_2_ has the dual properties of generating •OH and capturing electrons. An appropriate amount of H_2_O_2_ can be used as an electron trap to inhibit the recombination of hole electron pairs, produce strong oxidizing •OH, and promote the photocatalytic reaction. At the same time, H_2_O_2_ also consumed holes and •OH. The reduction in the number of holes and hydroxyl radicals inevitably reduced the photocatalytic reaction rate. When the concentration of H_2_O_2_ was high, the rate of H_2_O_2_ consumption of •OH exceeded the generation rate. Therefore, the excessive addition of H_2_O_2_ reduced the decolorization efficiency of MB solution by CuO/bentonite.

#### 3.3.2. Effect of Calcination Temperature on Decolorization Efficiency of MB Solution

**Figure 4:**
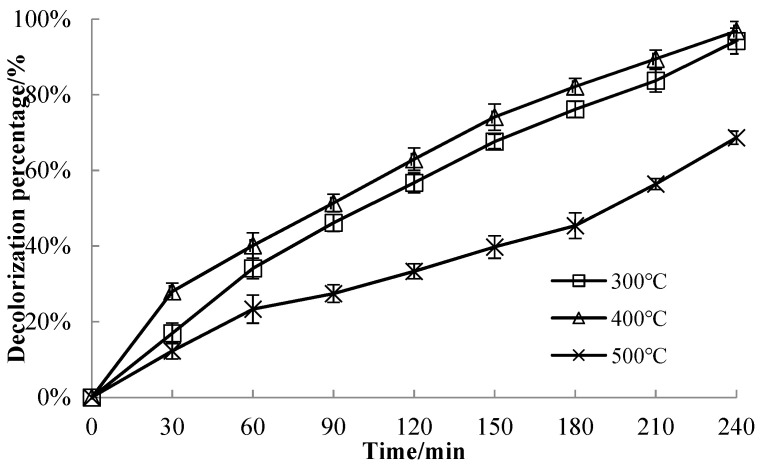
Effect of calcination temperature on the decolorizationefficiency of the MB solution.

The decolorization efficiency of a 50 mg/L MB solution by 1.4% CuO/bentonite composite at different calcination temperatures is shown in Figure 4. When the initial concentration of the MB solution was 50 mg/L, the pH value was 7.0, 1.5 mL H_2_O_2_ was added, the irradiation time was 240 min, the calcination temperature was 300 °C, 400 °C, and 500 °C, and the decolorization efficiency of CuO/bentonite on MB solution was 95.21%, 96.81%, 68.65%, respectively. It can be seen that, when the calcination temperature reached 400 °C, the photocatalyst had the highest catalytic activity. At a low calcination temperature, the proportion of CuO was lower, and part of the CuO existed in an amorphous state. Photogenerated electrons and holes were easy to compound; hence, the catalyst activity was low. It has been reported that Cu_2_O is generated at 300 °C, and CuO is not generated until the temperature increases to 400 °C [41]. With increasing calcination temperature, ion aggregation appeared on the surface of the CuO/bentonite, resulting in a decreasein specific surface area and a decreased decolorization efficiency of the MB solution. Therefore, 400 °C was selected as the calcination temperature.

#### 3.3.3. Effect of Catalyst Dosage on MB Solution Decolorization Performance

**Figure 5:**
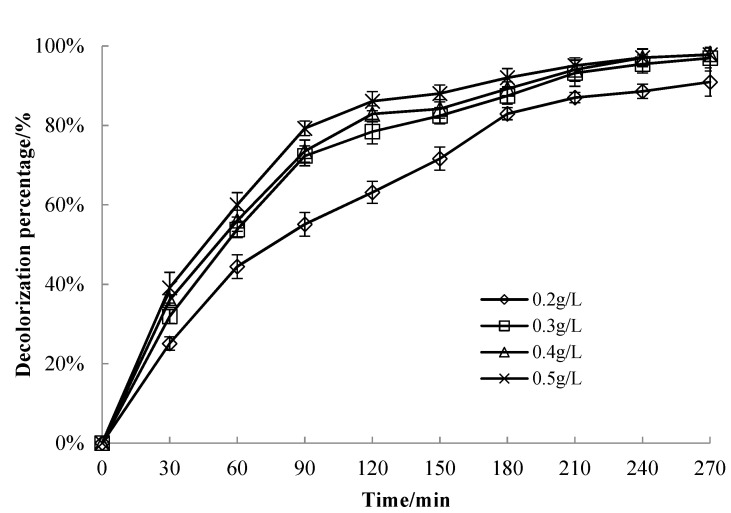
Effect of catalyst dosage on the degradation efficiency of the MB solution.

Four samples of 50 mg/L MB solution were taken, each being 50 mL with an adjusted pH value of 7.0. Subsequently, 1.4% CuO/bentonite (400 °C) composite was added in quantities of 0.2 g/L, 0.3 g/L, 0.4 g/L, and 0.5 g/L, and allowed to react in the dark for 30 min. Next, 1.5 mL of H_2_O_2_ was added and allowed to react at room temperature for 240 min under visible irradiation. The results are shown in Figure 5.

It can be seen from Figure 5 that, when the dosage of the catalysts was 0.3 g/L, 0.4 g/L, and 0.5 g/L, the decolorization efficiency of MB reached more than 96% after 240 min of irradiation. With an increase in the dosage, the decolorization efficiency increased at the end of the dark reaction. This indicates that bentonite’s adsorption played a significant role at this stage and then played a full role in the photocatalytic efficiency of CuO. Increasing the dosage of catalysts did not significantly improve photocatalytic efficiency. Hu studied the decolorization of methylene blue with iron-modified bentonite (Fe-B) as a heterogeneous Fenton catalyst with the same results as in this study. Their research also led to the conclusion that, with an increase in the dosage of catalyst, the main characteristics of surface active sites provided by Fe-B, the generation rate of hydroxyl radicals, and the decolorization efficiency of methylene blue were all increased. When the dosage of Fe-B reached 0.6 g/L, the decolorization efficiency of methylene blue was 98.23%. The decolorization efficiency of methylene blue was not significantly improved by increasing the dosage of Fe-B [42]. In summary, a catalyst dosage of 0.3 g/L was finally selected as optimal.

#### 3.3.4. Effect of Irradiation Time on Catalyst Performance

**Figure 6:**
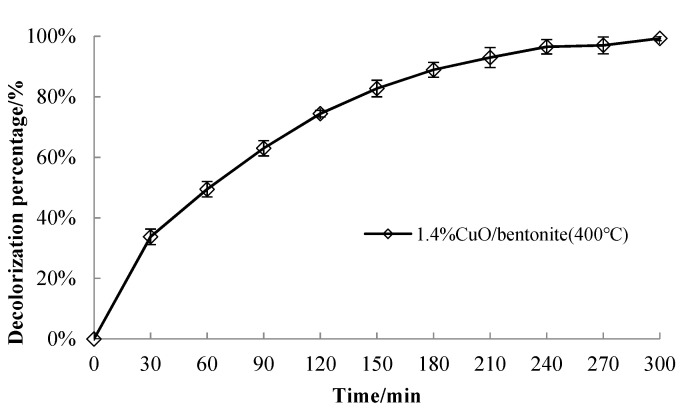
Effect of irradiation time on the catalytic activity of the catalyst.

A quantity of 50 mLof the 50 mg/L MB solution was taken and adjusted so that the pH value was 7.0. A quantity of 0.3 g/L of the 1.4% CuO/bentonite (400 °C) composite was added, and the reaction was conducted in the dark for 30 min. Subsequently, 1.5 mL of H_2_O_2_ was added to the 1.4% CuO/bentonite (400 °C) composite and allowed to react under visible irradiation at room temperature to investigate the effect of irradiation time on MB decolorization efficiency. The results are shown in Figure 6.

It can be seen from Figure 6 that, after 30 min of light avoidance, the decolorization efficiency of MB by the catalyst increased sharply from the beginning of visible irradiation to 180 min and then plateaued. This may be because the photocatalytic decolorization process of MB involved MB molecules being first adsorbed by the catalyst and then degraded. When the irradiation time was short, the adsorption rate of methylene blue on the catalyst was faster, and the surface decolorization was also faster. Subsequently, some molecules migrated and diffused to the pores and interlayers of the catalyst, and then photo-decolorization occurred. Some intermediates, which are difficult for OH radicals to oxidize, were formed during the decolorization process, resulting in low photo-decolorization efficiency and slow catalytic activity improvement. This is basically consistent with the results of Zhu on the photocatalytic decolorization of methyl orange by Cu-ZnO/bentonite [43]. However, the decolorization rate of methylene blue reached the maximum when exposed to visible irradiation for 240 min. According to the analysis presented above, it can be concludedthat the optimum irradiation time was 240 min.

#### 3.3.5. Effect of Solution pH on Catalyst Performance

**Figure 7:**
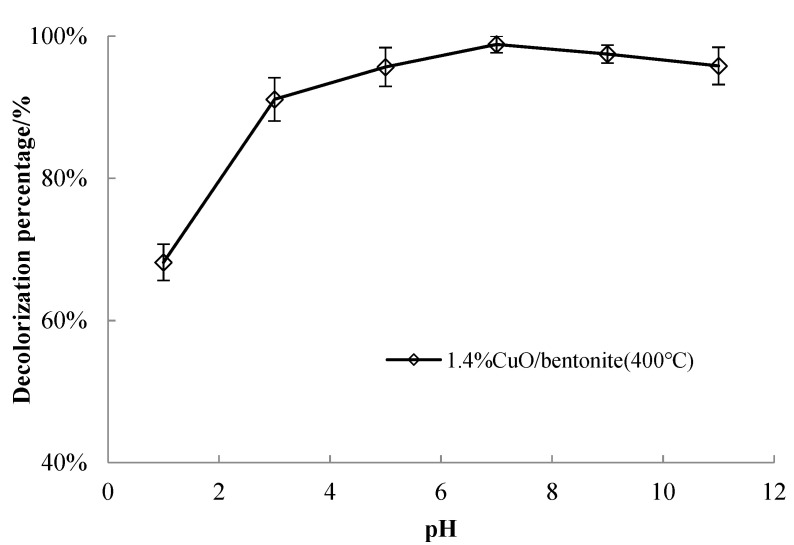
Effect of solution pH on catalyst performance.

Six 50 mL samples of the 50 mg/L MB solution were taken and adjusted for pH values of 1, 3, 5, 7, 9, and 11. A 0.3 g/L quantity of 1.4% CuO/bentonite (400 °C) photocatalyst was added to the MB solution for 30 min. Subsequently, 1.5 mL of H_2_O_2_ was added and allowed to react at room temperature for 240 min under visible irradiation. The effect of the initial pH value on MB decolorization efficiency was investigated, and the results are shown in Figure 7.

It can be seen from Figure 7 that, when the pH value of the solution was between 3 and 11, the decolorization efficiency of MB was higher, and the difference was not significant, being more than 90%. When the pH value was 7, the maximum decolorization efficiency of MB was 98.83%. The decolorization efficiency of MB decreased slightly with the increase in pH value. The reason may be that MB molecules compete with OH^−^ in the solution to adsorb onto the surface of the photocatalyst, which deteriorates under the degradation effect. CuO might react with acid and be deactivated under solid acid conditions. At the same time, the amount •OH generated by the direct interaction between holes and OH was reduced, which was not conducive to the degradation reaction. Compared with the literature on related catalysts, the obtained optimal pH of 7 is reasonable. For example, a study on the decolorization of MB by TiO_2_ nano sized particles [44] indicated that the photo-decolorization of MB increased with increasing pH from 3 to 11; that is, under medium and basic conditions, the decolorization process could occur in a wide range of pH. The optimal pH value of 7 was selected for other experiments. Thus, the initial pH value of MB was 7 for the photocatalytic response.

#### 3.3.6. Effect of Different Materials on the Decolorization Efficiency ofMB

**Figure 8:**
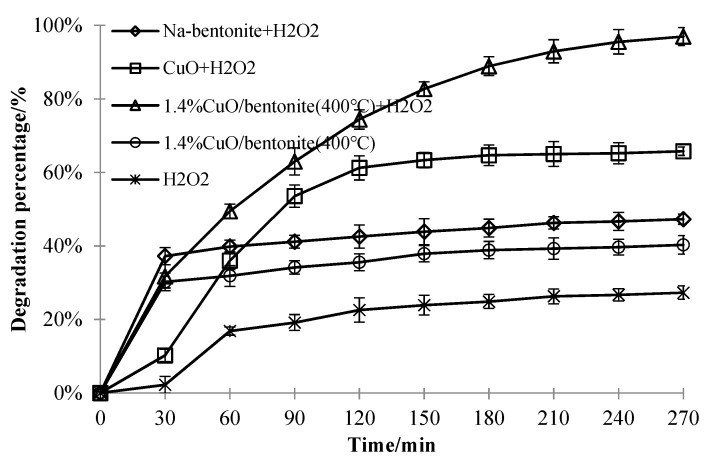
Effect of different catalysts on MB degradation performance.

To clarify the decolorization efficiency of methylene blue solution by different catalysts, the experimental conditions were as follows: the initial concentration of methylene blue solution was 50 mg/L, the pH value was 7.0, and the irradiation time was 240 min. Quantities of 0.3 g/L Na-bentonite, CuO, and 1.4% CuO/bentonite (400 °C) composite were added, and the reaction was conducted in the dark for 30 min; then, 1.5 mL of H_2_O_2_ was added to Na-bentonite, CuO and 1.4% CuO/bentonite (400 °C). The photocatalytic activity of the materials was determined by the decolorization efficiency of MB under visible irradiation. Furthermore, the activity of 1.4% CuO/bentonite (400 °C) could be demonstrated by comparison. The results are shown in Figure 8.

After considering the adsorption of materials, it can be seen from Figure 8 that the 1.4% CuO/bentonite (400 °C) composite degraded 49.7% of the MB. Under dark reaction conditions, the decolorization efficiency of methylene blue by Na-bentonite was significantly higher than that by CuO, 1.4% CuO/bentonite (400 °C), and H_2_O_2_. This decolorization was mainly due to the adsorption of dye molecules by bentonite. After the CuO loading on bentonite, the specific surface area of 1.4% CuO/bentonite (400 °C) (S_BET_ = 96.1 m^2^/g) decreased slightly, which shows that the introduction of CuO had little effect on the specific surface area of Na-bentonite (S_BET_ = 98.6 m^2^/g). The decolorization efficiency of 1.4% CuO/bentonite (400 °C) for the MB solution that was lower than that of Na-bentonite, which may be due to the CuO loading onto the bentonite-occupied part of the adsorption sites. When H_2_O_2_ was added to the solution with the catalyst containing 1.4% CuO/bentonite (400 °C), the decolorization of MB was significantly higher than that of Na-bentonite, CuO, and 1.4% CuO/bentonite (400 °C) without the addition of H_2_O_2_ within the same irradiation time. H_2_O_2_ can produce strong oxidizing •OH and promote the photocatalytic reaction. The addition of H_2_O_2_ was necessary for improving the photocatalytic activity of CuO/bentonite. The decolorization efficiency of 1.4% CuO/bentonite (400 °C) was 31.22% higher than that of CuO, and the combined action of CuO photocatalysis and bentonite adsorption improved the decolorization efficiency of 1.4% CuO/bentonite (400 °C) for MB. Guo studied the effect of a Ce-doped ZnO/bentonite composite photocatalytic material on the decolorization efficiency of MB. The decolorization efficiency of MB by the bentonite composite photocatalytic material loaded with pure ZnO for 2 h was 81.1%, which is 15.88% lower than that obtained in this study [45].

#### 3.3.7. Adsorption Kinetics

**Figure 9:**
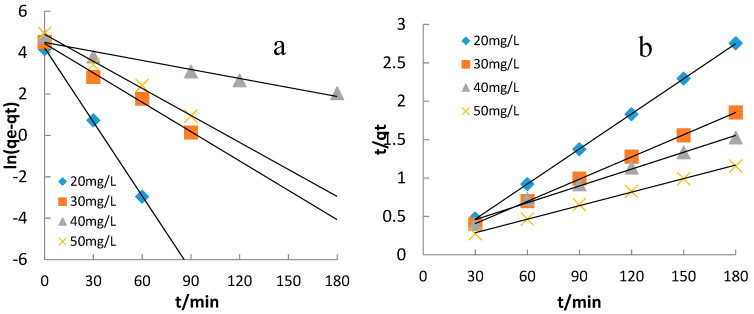
Pseudo-first-order kineticcurve (**a**) and pseudo-second-order kinetic curve (**b**) of photocatalytic degradation of MB by 1.4% CuO/bentonite (400 °C) composite at different MB concentrations.

The pseudo-first-order kinetic model assumes that diffusion steps control the adsorption rate. In contrast, the pseudo-second-order kinetic model assumes that the adsorption rate is controlled by a chemical mechanism [35]. The pseudo-first-order kinetic curve (a) and pseudo-second-order kinetic curve (b) of the 1.4% CuO/bentonite (400 °C) composite for MB adsorption at different MB concentrations are shown in Figure 9.

Compared with the pseudo-first-order kinetic model, the fitting degree of all the pseudo-second-order kinetic model data points at different MB concentrations was relatively high (Table 1). Thus, this model was used to determine the adsorption process occurring in the photocatalytic degradation of MB by the 1.4% CuO/bentonite (400 °C) composite. It can be seen from Figure 9 that the graphical representation of the pseudo-second-order dynamic model was almost linear in form, demonstrating the perfect fit of the model to this process. The pseudo-second-order kinetic model better fit the kinetic adsorption process of 1.4% CuO/bentonite (400 °C) composite for MB. The pseudo-second-order dynamic model better fit the process under investigation; thus, a chemical mechanism controlled the adsorption rate of 1.4% CuO/bentonite (400 °C) composite for MB. Szostak and Banach from the Faculty of Chemical Engineering and Technology in Cracow, Poland, studied the kinetics of sorption and photocatalytic degradation of methylene blue photocatalysis onto a bentonite–ZnO–CuO nanocomposite and obtained similar results [23].

#### 3.3.8. Recycling of Catalyst

**Figure 10:**
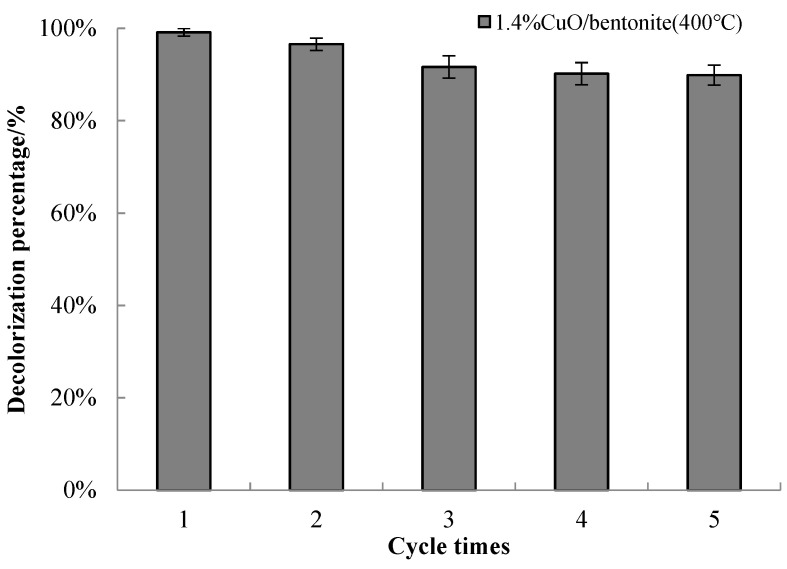
Reuse effect of 1.4% CuO/bentonite (400 °C) composite.

The reuse of catalysts plays a vital role in the practical application of photocatalysts. Therefore, the used catalyst was reused and evaluated. The experimental conditions were as follows: 1.4% CuO/bentonite (400 °C) of the used catalyst was washed with water, dried at 80 °C, and calcined at 400 °C for regeneration. Subsequently, the regenerated 1.4% CuO/bentonite (400 °C) composite was used for photocatalytic degradation of the 50 mg/L MB solution under visible irradiation. The pH value of the solution was adjusted to 7.0, and the irradiation reaction was carried out at room temperature for 240 min. The composite was recycled five times, and the decolorization efficiency was reasonable. The results are shown in Figure 10.

It can be seen from Figure 10 that the 1.4% CuO/bentonite (400 °C) composite still had a high photocatalytic activity after five cycles of degradation, and the decolorization efficiency was close to 90%. These results imply that the material had excellent reusability and regeneration performance. According to Szostak, the q_max_ parameter had a higher value when looking at the sorption and photocatalytic degradation of methylene blue on a bentonite–ZnO–CuO nanocomposite, as well as a higher concentration (100 mg/L) for the combined photocatalytic–sorption process than for sorption itself. This shows that photodegradation occurred during the process [23]. Cui recycled CuO nanoparticles/zeolite six times to degrade MB, and the decolorization efficiency only decreased from 95% to 92.4%, which is comparable to this study, although this study needed a longer irradiation time [27]. The regeneration performance was adequate, which allows effectively reducing the cost of wastewater treatment. In summary, the 1.4% CuO/bentonite (400 °C) composite photocatalyst showed good reusability and stability.

### 3.4. Photocatalytic Mechanism ofCuO/Bentonite Composite

**Figure 11:**
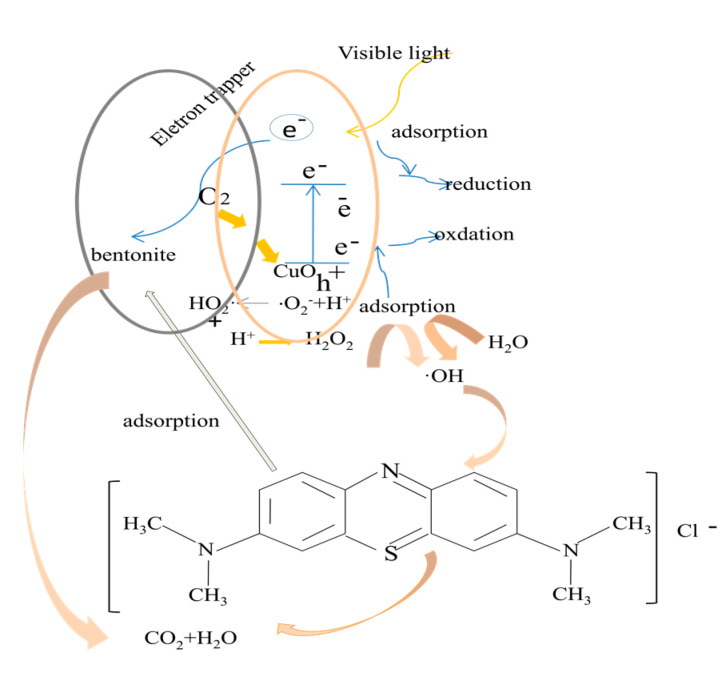
Photocatalytic degradation mechanism diagram of CuO/bentonite composite.

The photocatalytic degradation mechanism of CuO/bentonite composite is shown in Figure 11. It can be seen from Figure 11 that, on the one hand, the decolorization process of MB solution by the CuO/bentonite composite is partly due to the adsorption of bentonite; on the other hand, it is also partly due to the photocatalytic effect of CuO under visible irradiation.

It can be seen from Figure 11 that, when the CuO semiconductor surface is visibly irradiated, the valence band electrons have inter-band transitions, resulting in photogenerated electrons (e^−^) and holes (h^+^), and the generated electrons and holes are quickly transferred to the surface of the catalyst through CuO. Photogenerated holes can directly oxidize MB molecules that adsorb onto the surface of CuO/bentonite photocatalyst, resulting in dye decolorization. Photogenerated electrons can be accepted by dissolved oxygen or participate in the reduction of water to separate photogenerated electron–hole pairs. Additionally, OH^−^ can react with water to form hydroxyl radicals (•OH) and indirectly lead to strong oxidation of MB. Equation (10) shows that the equilibrium between H^+^ and •O_2_^−^ leads to the formation of the HO_2_• and leads to the production of H_2_O_2_ in Equation (11) [27].

In order to improve the photocatalytic activity of CuO/bentonite, H_2_O_2_ needs to be added. In many studies, H_2_O_2_ has been used as an additive to enhance the photocatalytic activity of semiconductors, such as TiO_2_ [46] and ZnO [47]. The reason is that H_2_O_2_ is a good electron acceptor, which is converted into •OH after receiving electrons. The hydroxyl radical has strong oxidation ability and can degrade organic pollutants into CO_2_ and H_2_O, which plays an important role in the photocatalytic reaction [48]. The CuO/bentonite photocatalyst can be used to degrade MB in water to decolorize the solution, leading to the following response:

CuO/bentonite + hυ (≥energy gap) → CuO/bentonite (e^−^ + h^+^), (7)

e^−^ + O_2_ → •O_2_^−^, (8)

(H_2_O↔ H^+^ + OH^−^) + h^+^ → H^+^ + •OH, (9)

H^+^ + •O_2_^−^ → HO_2_•, (10)

HO_2_• + H^+^ → H_2_O_2_, (11)

H_2_O_2_ + e^−^ → OH^−^ + •OH, (12)

MB + h^+^ → oxidation of MB, (13)

MB + e^−^ → reductionof MB, (14)

•OH + MB → CO_2_ + H_2_O, (15)

•O_2_^−^ + MB → CO_2_ + H_2_O. (16)

## 4. Conclusions

A new type of CuO/bentonite composite photocatalyst was prepared on the basis of the dual effect of the adsorption capacity of bentonite and the photocatalytic activity of CuO. Copper existed as CuO in the composite. The degradation conditions were optimized as a pH of 7.0, catalyst dosage of 0.3 g/L, H_2_O_2_ dosage of 1.5 mL, and initial MB concentration of 50 mg/L, reacted at room temperature for 240 min under visible irradiation. Under the optimum conditions, the decolorization efficiency of 1.4% CuO/bentonite (400 °C) composite reached 96.98% after 240 min of visible irradiation, which was significantly higher than that of CuO and Na-bentonite. The decolorization efficiency of 1.4% CuO/bentonite (400 °C) was nearly 90% after being recycled five times. Thus, CuO/bentonite exhibited excellent reusability and regeneration performance. Among the two investigated kinetic models, the pseudo-second-order model best suited the sorption with simultaneous photocatalysis. This composite structure will provide new ideas and research directions for the circulation and aggregation of this photocatalyst in water.

## Figures and Tables

**Table 1:** Parameters of kinetic models for MB photocatalysison 1.4% CuO/bentonite (400 °C) composite.

MBConcentration (mg/L)	Pseudo-First Order			Pseudo-Second Order		
	q_e_ (mg/g)	k_1_ (min^−1^)	*R^2^*	q_e_ (mg/g)	k_2_ (g·mg^−1^·min^−1^)	*R^2^*
20	68.03	0.119	0.998	66.67	0.015	0.999
30	84.85	0.047	0.992	111.11	0.009	0.999
40	89.74	0.014	0.957	142.86	0.007	0.991
50	132.42	0.043	0.994	200.00	0.005	0.998
